# Genetic diagnostic features after failure of initial treatment with epidermal growth factor receptor (EGFR)-tyrosine kinase inhibitors among non-small-cell lung cancer patients harboring *EGFR* mutations

**DOI:** 10.1186/s12885-020-07424-w

**Published:** 2020-10-02

**Authors:** Yuichiro Takeda, Go Naka, Yoh Yamaguchi, Masao Hashimoto, Manabu Suzuki, Shinyu Izumi, Haruhito Sugiyama

**Affiliations:** Department of Respiratory Medicine, National Center for Global Health and Medicine, 1-21-1 Toyama, Shinjuku-ku, Tokyo, 162-8655 Japan

**Keywords:** T790M, Repeated biopsy, Single-plexus PCR, EGFR-TKI

## Abstract

**Background:**

Osimertinib, a third-generation epidermal growth factor receptor (EGFR)-tyrosine kinase inhibitor (TKI), can be used as second-line treatment for lung cancer patients harboring the T790M substitution. Although osimertinib is more effective than the first-generation EGFR-TKIs used for first-line treatment, its efficacy with respect to long-term patient survival remains unclear even upon the administration of a complete sequence of EGFR-TKI therapy. Moreover, limited information is available regarding genetic diagnostic approaches after the treatment of EGFR-TKI–naïve patients. This study investigated the clinical characteristics of *EGFR*-mutated lung cancer patients harboring the T790M substitution resistant to EGFR-TKIs, as well as the advantages of rebiopsy and liquid biopsy for these patients.

**Methods:**

The medical records of patients screened for *EGFR* mutations were reviewed. Upon failure of naïve treatment with EGFR-TKIs, except for osimertinib, single-plexus cobas version 2 was repeatedly used to detect the T790M substitution in *EGFR* via tissue or liquid biopsy.

**Results:**

From April 2016 through May 2019, 113 patients were found to harbor *EGFR* mutations. Sixty patients were treated with EGFR-TKIs, among whom 46 underwent tissue or liquid biopsy. Twenty-nine of these 46 (63%) patients harbored the T790M substitution. In total, 141 rebiopsies were performed. The T790M substitution was detected in 24 of 43 tissue biopsies and 11 of 98 liquid biopsies. If patients displayed an *EGFR* exon 19 deletion, had a new lesion, and were administered gefitinib as first-line therapy, they were suspected to harbor the T790M substitution. Furthermore, the T790M substitution was detected through rebiopsy in patients with coexisting original mutations, brain metastases, tumor enlargement by ≥12 mm, or metastases at minor sites.

**Conclusion:**

Among patients with positive factors associated with the T790M mutation, repeated tissue or liquid biopsies are useful to maximize the detection rate of the T790M substitution. Furthermore, these biopsies need to be repeated numerous times in order to reduce “detection overlook” among such patients.

## Key message

*Three alternatives are currently available for the use of first- or second-generation epidermal growth factor receptor tyrosine kinase inhibitors (EGFR-TKIs) to treat EGFR-mutated lung cancer, such as single agents or combinatorial treatment with an anti-VEGF antibody or chemotherapy. After the failure of initial EGFR-TKI treatment, repeated biopsy is expected to maximize the detection of T790M substitutions, thus prompting osimertinib therapy. Patients with positive factors associated with the T790M mutation would benefit from biopsies repeated numerous times.*

## Background

Patients with metastatic non-small-cell lung cancer (NSCLC) harboring epidermal growth factor receptor (EGFR)-sensitizing mutations generally receive EGFR-tyrosine kinase inhibitors (TKIs) as the first-line treatment [1]. Five TKIs, including first- to third-generation TKIs, are available for EGFR-TKI therapy. Although most patients eventually become resistant to EGFR-TKIs, the *EGFR* p.Thr790Met point mutation (*EGFR* T790M) is detected in 30–50% of patients presenting with disease progression after receiving first- or second-generation TKIs [2, 3]. These patients can be treated with osimertinib, whereas other patients might be treated with cytotoxic chemotherapy. Although osimertinib is generally preferred as first-line therapy because of efficacy and tolerability [4], patients with disease progression upon osimertinib treatment have been administered only cytotoxic chemotherapy [5]. Recent studies have revealed numerous EGFR-TKI–based alternatives for first-line treatment. First-generation EGFR-TKIs have been used in combination with an anti-VEGF antibody or chemotherapy [6–8]. These clinical trials reported almost the same high efficacy as that of osimertinib, despite slightly increased toxicities. In these trials, the *EGFR* T790M substitution was also suspected in ≥50% of patients presenting with disease progression. Furthermore, second-generation EGFR-TKIs constitute first-line treatment alternatives for *EGFR*-mutated advanced NSCLC because no phase III clinical trial has compared the clinical efficacy of second-generation EGFR-TKIs and osimertinib. When NSCLC patients harboring *EGFR* mutations are administered EGFR-TKIs except for osimertinib as first-line treatment, approximately half of them qualify for osimertinib therapy. For second- or third-line treatment of patients with osimertinib to maximize the treatment duration for EGFR-TKIs since April 2018 [9], it is essential to detect the T790M substitution maximally. Cobas ver. 2 can be used for companion diagnostic examination (CDx) [10]. Limited information is available on maximizing the detection of the T790M substitution using this type of CDx. Repeated rebiopsy is considered more effective in reducing “detection overlook” of the T790M mutation when rebiopsy is performed for patients with this mutation and disease progression with clinical features of the T790M substitution. The purpose of this study was to investigate the clinical characteristics of *EGFR*-mutated lung cancer patients harboring the T790M substitution that were resistant to EGFR-TKIs. Through this knowledge, we will be able to identify appropriate patients who require repeated tissue or liquid rebiopsy.

## Methods

### Patients

From April 2016 to May 2019, consecutive patients screened for *EGFR* mutations were retrospectively reviewed at the National Center for Global Health and Medicine, Japan. The peptide nucleic acid-locked nucleic acid (PNA-LNA) PCR clamp method [11] was used to detect *EGFR* mutations, using tissue biopsy specimens during the initial diagnosis of non-small non-squamous-cell lung cancer. After lung cancer acquired clinical resistance to EGFR-TKIs, the cobas® EGFR Mutation Test (Version 2; Roche Molecular Systems) [10] was repeatedly performed to detect T790M mutation status through tissue or liquid biopsy. Clinical resistance was defined as an increase in the monitoring of tumor markers, disease progression through radiological imaging, or clinical disease progression.

### Rebiopsy and genetic analysis

All types of clinical rebiopsies were repeated when patients were suspected to be clinically resistant to EGFR-TKIs. If patients were likely to provide tumor tissue through a clinical procedure (e.g., bronchoscopy or computed tomography (CT)-guided biopsy) at radiographic disease progression, they underwent tissue biopsy numerous times. Otherwise, liquid biopsy was performed. After each rebiopsy, cobas® version 2 was used. When a new T790M substitution was detected, patients were administered osimertinib; if not, they were administered treatment other than osimertinib, such as cytotoxic chemotherapy or other molecular-targeted therapy. Tissue or liquid rebiopsies were repeated numerous times until the T790M substitution was detected. Cobas® version 2 is a single-plexus real-time PCR procedure to detect EGFR mutations, using unstained 5 μm thick sections obtained from a formalin-fixed paraffin-embed block and mounted on slides or whole-blood samples, as previously reported [10]. Mutations were analyzed at the central laboratory of LSI Medience Corporation (Tokyo, Japan).

### Data collection

The following data were obtained from each patient’s medical records: patient characteristics, including age, sex, smoking index, smoking status, comorbidities, and Eastern Cooperative Oncology Group performance status at diagnosis; oncological data, including histologic type, staging in accordance with the 8th edition of the TNM Classification of Malignant Tumors [12], tumor size of biopsy site, number of tumor lesions, metastatic organ, and *EGFR* mutation sites detected via the PNA-LNA PCR clamp method or cobas® version 2; treatment data, including surgical treatment, radiotherapy including radical or palliative radiation, and pharmacotherapy (gefitinib, erlotinib, afatinib, and osimertinib) for EGFR-TKI–naïve lines; subsequent systemic therapies, including cytotoxic chemotherapy regimens, immunotherapy, or other molecular-targeted treatment; data on the best supportive care; and tumor markers for CEA (ng/mL). CT, positron-emission CT, and magnetic resolution imaging were performed within 1 month of each biopsy for corresponding biopsy specimens. Patients harboring the T790M substitution were defined under the category of “detection of at least one T790M using single-plexus PCR through any type of clinically available biopsy.”

### Ethical considerations

The study was conducted in accordance with the tenets of the Declaration of Helsinki. The study was approved by the certified review board of the National Center for Global Health and Medicine (NCGM-G-003361-00). In accordance with the Japanese Ethical Guidelines for Medical and Health Research Involving Human Subjects, we used the opt-out method. We informed the participants about this study and obtained informed consent from subjects by displaying the disclosure document in the hospital as per the approval date until January 31, 2020.

### Statistical analysis

The primary outcome was the identification of clinical characteristics of *EGFR*-mutated lung cancer patients harboring the T790M substitution with acquired clinical resistance to EGFR-TKIs. Secondary outcomes included identifying factors inducing the T790M substitution through any type of rebiopsy among patients harboring the T790M substitution and factors through liquid rebiopsy. Fisher’s exact test was performed to compare the proportion of subjects with dichotomous outcomes in both groups. We used some dichotomous variables from original continuous variables to be suitable for the logistic regression model. The optimal cutoff values of each continuous variable were set by receiver-operator characteristic (ROC) curves by SigmaPlot version 14 software (Systat Software, Inc., San Jose, CA, USA). At a *p*-value of < 0.05, the optimal cutoff values of these continuous variables were set on the basis of a pre-test probability of 0.5 and a cost ratio of 1.0.

Because logistic regression analysis can determine the strength of association between each factor and the outcome, this analysis was performed to assess the aforementioned three factors, as previously described [13]. To select a multivariate analysis model, we identified variables with a *p*-value of less than 0.15 based on univariate analysis. Spearman’s rank test and clinically clarified dependent variables were used to exclude dependent variables from the aforementioned selected variables. A correlation coefficient (ρ) of more than 0.3 as the absolute value based on Spearman’s rank test indicated a significant association. Some models were constructed with only independent variables as candidates. ROC curves were used to select the best model among candidate models. In the final multivariate analysis using the simultaneous method, statistical significance was determined at *p* <  0.05 through a two-sided test. All analyses were performed using SPSS Statistics software version 25 (IBM, Armonk, NY, USA) or Stata version 15.1 (StataCorp LLC, College Station, TX, USA).

## Results

### Patients

**Fig. 1:**
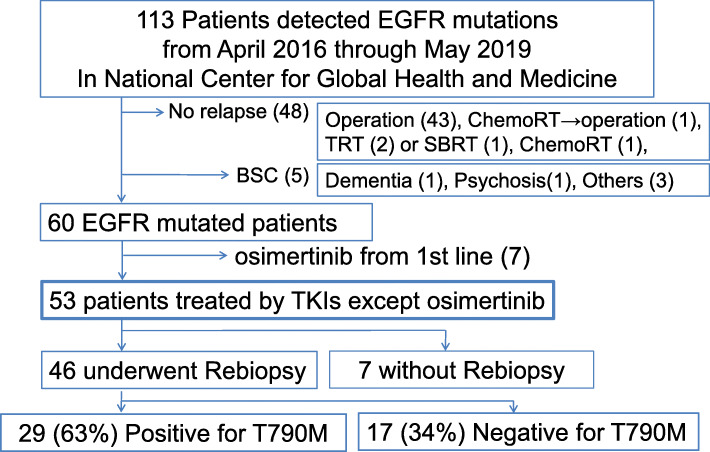
Study cohort. The data are the number of patients, unless specified otherwise. The thick framed square indicates the full analysis set of this study

Among 405 consecutively examined patients, *EGFR* mutations were detected in 113 patients (Fig. 1). Five patients decided to undergo only the best supportive care, and 48 did not experience relapse after local therapy, including surgery, radiotherapy, or chemoradiotherapy. Sixty patients harbored activating *EGFR* mutations, and seven were administered osimertinib as TKI-naïve therapy. The remaining patients were treated with TKIs, except for osimertinib. The T790M substitution was detected in 29 of 46 (63%) patients who underwent rebiopsy. Table 1 outlines the demographic characteristics of each group. Thirty-one patients presented with postoperative recurrence, and four patients presented with post-irradiation recurrence. During the overall study period, 33 patients received chemotherapy. During TKI-naïve treatment, 13 patients received gefitinib, 32 received erlotinib, and eight received afatinib.

**Table 1:** Patient background characteristics (*n* = 53)

Variable	Rebiopsy		
	(+)		(−)
	T790M mutation		
	Positive (*n* = 29)	Negative (*n* = 17)	Unknown (*n* = 7)
Sex			
Male	11	3	3
Female	18	14	4
Age (years) median (range)	74 (42 - 86)	72 (38 - 89)	70 (59 - 82)
Histology			
Adenocarcinoma	27	17	7
others	2	0	0
EGFR mutation at initial diagnosis			
Exon 19 deletion	21	6	1
L858R	8	9	5
L861Q	0	1	0
Exon 20 insertion	0	1	0
Compound mutation	0	0	1
Smoking status			
Never	18	13	3
Past	8	1	2
Current	3	3	2
ECOG performance status			
0	20	12	3
1	8	5	2
2	1	0	1
3	0	0	1
Stage at initial diagnosis			
IA - IIIC	10	3	3
IVA - IVB	19	14	4
Surgical treatment			
No	13	6	3
Yes	16	11	4
Radical Radiotherapy			
No	27	16	6
Yes	2	1	1
Chemotherapy during treatment			
No	8	6	6
Yes	21	11	1
Reason for termination of TKIs			
Ongoing	1	5	3
Adverse events	0	1	3
Tumor growth	14	3	0
New lesions	14	8	1
“CEA at progression” / “nadir CEA”; median (range)	3.1 (1.1–170)	2.5 (1.3–7.88)	2.0 (0.5–3.57)
nadir CEA (ng/mL); median (range)	3.0 (0.8–36.9)	2.2 (1.0–78.9)	45.6 (1.4–198)
Medical Drugs at TKI naïve line			
Gefitinib	11	2	0
Erlotinib	16	10	6
Afatinib	2	5	1

Abbreviations: *ECOG* Eastern Cooperative Oncology Group; *TKI* tyrosine kinase inhibitor; *CEA* carcinoembryonic antigen; *“CEA at progression” / “nadir CEA”* “Serum level of CEA at progression” divided by “Serum nadir level of CEA”; *nadir CEA* Serum nadir level of CEA

### Rebiopsy outcomes

To identify patients harboring the T790M substitution, all types of clinically available rebiopsies were performed numerous times (Table 2). Tissue biopsy was repeated a maximum of four times. During the initial tissue biopsy, the detection rate (DR) was 67.8%, with a sensitivity of 80.8%. After that, the DR was approximately 30%, with a cumulative sensitivity (CS) of 77.4–79.3%. In terms of liquid biopsy, the maximum frequency of rebiopsy was 10. During the initial liquid biopsy, the DR was 8.1%, with a sensitivity of 13%. During each liquid biopsy, the median DR was 8.6%, ranging from 0 to 25%, and the median cumulative sensitivity was 18.9%, ranging from 16.7 to 20.6%. In total, we performed 141 rebiopsies, including both tissue and liquid biopsies, from 46 patients (Table 3). Among these patients, 29 (63%) harbored the T790M substitution. The T790M substitution was detected in 35 biopsies, with a CS of 39.3%, including 24 tissue biopsies with 77.4% and 11 liquid biopsies with 19%, respectively. Regarding the detection of the T790M substitution, significant differences between tissue and liquid biopsy were observed based on Fisher’s exact test (*P* <  0.0001).

**Table 2:** Number of biopsy and detection on T790M

	1st	2nd	3rd	4th							Total
**Tissue biopsy (Individual test)**											
**T790M positive**	**21**	**2**	**0**	**1**							**24**
With original mutation	26	5	3	3							37
**Total**	**31**	**6**	**3**	**3**							**43**
**Tissue biopsy (Cumulative test)**											
**T790M positive**	**21**	**23**	**23**	**24**							**24**
Cumulative sensitivity	0.81	0.79	0.77	0.77							0.77
**Total**	**31**	**37**	**40**	**43**							**43**
	**1st**	**2nd**	**3rd**	**4th**	**5th**	**6th**	**7th**	**8th**	**9th**	**10th**	**Total**
**Liquid biopsy (Individual test)**											
**T790M positive**	**3**	**4**	**1**	**0**	**0**	**1**	**1**	**1**	**0**	**0**	**11**
With original mutation	13	5	4	1	1	2	1	1	0	0	28
**Total**	**37**	**17**	**11**	**7**	**7**	**7**	**5**	**4**	**2**	**1**	**98**
**Liquid biopsy (Cumulative test)**											
**T790M positive**	**3**	**7**	**8**	**8**	**8**	**9**	**10**	**11**	**11**	**11**	**11**
Cumulative sensitivity	0.13	0.21	0.2	0.18	0.17	0.17	0.18	0.19	0.19	0.19	0.19
**Total**	**37**	**54**	**65**	**72**	**79**	**86**	**91**	**95**	**97**	**98**	**98**

**Table 3:** Rebiopsy outcomes

Variable	T790M mutation		
	Positive patients (*n* = 29)	Negative patients (n = 17)	Total patients (*n* = 46)
**Tissue biopsy (included cytology) count**	**31**	**12**	**43**
**T790M positive count**	**24 (77.4)**	**–**	**24 (55.8)**
With Original mutation	22	–	22
Without Original mutation	2	–	2
**T790M negative count**	**7 (22.6)**	**12**	**19 (44.2)**
With Original mutation	5	10	15
Without Original mutation	2	2	4
**Liquid biopsy count**	**58**	**40**	**98**
**T790M positive count**	**11 (19.0)**	**–**	**11 (11.2)**
With Original mutation	10	–	10
Without Original mutation	1	–	1
**T790M negative count**	**47 (81.0)**	**40**	**87 (88.8)**
With Original mutation	9	11	20
Without Original mutation	38	29	67
**Total rebiopsy count**	**89**	**52**	**141**

Data are number of patients (%) unless specified otherwise

### Positive clinical factors in patients harboring the T790M substitution

Our main purpose was to elucidate clinical features of positivity for the T790M substitution through clinically available mutational analysis. We considered the target patients with rebiopsy as 53 patients treated with TKIs except osimertinib, comprising the full analysis set (Fig. 1). Because logistic regression analysis can identify the strength of association between each clinical factor and the primary outcome, we analyzed 53 patients treated with EGFR-TKIs other than osimertinib. The results of logistic regression analyses are shown in Table 4. Six variables had *p*-values  < 0.15 based on univariate analyses. Multivariate analysis indicated that significant clinical features associated with patients harboring the T790M substitution were as follows: exon 19 deletions in the original mutation, termination of TKIs owing to the detection of new lesions, and gefitinib in TKI-naïve treatment.

**Table 4:** Logistic regression analysis to identify patients harboring the T790M substitution among those with EGFR-mutated lung cancer (n = 53)

Variables	Univariate Analyses			Multivariate Analysis		
	OR	95%CI	*P-value*	OR	95%CI	*P-value*
**Mutation site at initial diagnosis; Exon 19 Deletion vs. L858R**	**0.17**	**0.05–0.51**	**0.002**	**0.04**	**0.004–0.34**	**0.003**
**Reason for termination of TKIs;**	**2.26**	**1.25–4.11**	**0.007**	**3.65**	**1.39–9.59**	**0.008**
*Ongoing*	*Reference*			*Reference*		
*AEs*	*1*	**–**	**–**	*1*	**–**	**–**
*Tumor growth*	*37.3*	*3.30–421.6*	*0.003*	*33.8*	*1.60–713.3*	*0.024*
*New lesions*	*12.4*	*1.32–117.0*	*0.027*	*44.9*	*2.13–950.9*	*0.014*
**Medical Drugs at TKI-naïve line**	**0.24**	**0.08–0.72**	**0.01**	**0.09**	**0.01–0.62**	**0.014**
*Gefitinib*	*Reference*			*Reference*		
*Erlotinib*	*0.18*	*0.035–0.95*	*0.044*	*0.49*	*0.02–0.99*	*0.049*
*Afatinib*	*0.06*	*0.007–0.55*	*0.012*	*0.01*	*0.0002–0.53*	*0.02*
Chemotherapy; absent vs. present	2.63	0.84–8.22	0.09	NI		
“Serum level of CEA at progression” divided by “Serum nadir level of CEA”	1.16	0.97–1.39	0.11	1.22	0.77–1.93	0.4
Serum nadir level of CEA (ng/mL)	0.97	0.95–1.01	0.14	NI		

Abbreviations: *OR* Odds ratio; *CI* confidence interval; *TKI* tyrosine kinase inhibitor; *AEs* adverse effects; *CEA* carcinoembryonic antigen; Variables with a p-value < 0.15 on univariate analysis were entered into multivariate logistical analysis by a simultaneous method. NI, not included in the best multivariate logistic regression model

### Positive factors associated with the T790M substitution upon rebiopsy

Because logistic regression analysis can find the degree of association between each clinical factor and one of the secondary outcomes, 89 rebiopsies were performed among patients harboring this mutation. Table 5 shows the background characteristics of patients harboring the T790M substitution upon rebiopsy. Logistic regression analyses revealed 16 variables with *p*-values < 0.15 based on univariate analyses. We constructed four sets of multivariate models comprising variables that were not correlated with each other, as follows. Model 1 consisted of five variables, i.e., original mutation, brain metastases, enlargement of tumor size, mutation site at initial diagnosis, or minor site metastases, and the AUC was 0.916. Model 2 consisted of original mutation, enlargement of tumor size, mutation site at initial diagnosis, minor site metastases, or new brain metastases, and the AUC was 0.911. Model 3 comprised variables, i.e., type of biopsy, brain metastases, mutation site at initial diagnosis, or minor site metastases, and the AUC was 0.881. Model 4 comprised brain metastases, mutation site at initial diagnosis, enlargement of tumor size, or detection of new tumor lesion, with an AUC of 0.824. The best model selected through ROC curve analysis is Model 1, as shown in Table 6. Multivariate analysis showed that the significant associations detected with the T790M substitution upon rebiopsy among patients harboring T790M were as follows: co-detection of the original mutation, co-occurring brain metastases, tumor enlargement of ≥12 mm, or involvement of minor site metastases, which includes metastases of the skin, kidney, adrenal glands among other organs, as well as ascites and lymphangiosis carcinomatosa.

**Table 5:** Background characteristics on patients harboring the T790M substitution upon rebiopsy (*n* = 89)

Variable	T790M mutation		
	Positive (*n* = 35)	Negative (*n* = 54)	Total
Original mutation			
Absent	3	40	43
Present	32	14	46
Type of Biopsy;			
Liquid	11	47	58
Tissue	24	7	31
Detection of New tumor lesion;			
< 4	11	39	50
4 ≤	24	15	39
Number of Tissue biopsy			
Median (Range)	1 (0–4)	0 (0–3)	0 (0–4)
Detection of New metastatic organ, *n* = 88			
0	12	39	51
1	12	10	22
2	4	4	8
3	3	1	4
4	3	0	3
Number of tumor lesion			
< 6	6	29	35
6 ≤	29	25	54
Brain metastases, *n* = 88			
Absent	16	43	59
Present	18	11	29
Bone metastases, n = 88			
Absent	26	42	68
Present	8	12	20
Number of Liquid biopsy			
Median (Range)	0 (0–8)	2 (0–9)	1 (0–9)
Enlargement of Tumor size			
< 12 mm	15	39	54
12 mm ≤	20	15	35
New brain metastases, n = 88			
Absent	25	50	75
Present	9	4	13
Mutation site at initial diagnosis			
Exon19 Deletion	24	47	71
L858R	11	7	18
Minor site metastases, n = 88			
Absent	27	51	78
Present	7	3	10
New hepatic metastases, n = 88			
Absent	28	51	79
Present	6	3	9
New minor site metastases, n = 88			
Absent	29	52	81
Present	5	2	7
Hepatic metastases, n = 88			
Absent	27	49	76
Present	7	5	12

Abbreviations: n; number, minor site metastases; metastases of skin, kidney, ascites, lymphangiosis carcinomatosa, adrenal organ or others,

**Table 6:** Logistic regression analysis to identify patients harboring the T790M substitution upon rebiopsy (n = 89)

Variables	Univariate Analyses			Multivariate Analysis		
	OR	95%CI	*P-value*	OR	95%CI	*P-value*
**Original mutation; absent vs. present**	**30.5**	**8.05–115.3**	**< 0.001**	**41.5**	**6.53–264.3**	**< 0.001**
Type of Biopsy; Liquid vs. Tissue	14.6	5.04–42.6	< 0.001	NI		
Detection of New tumor lesion; < 4 vs 4 ≤	5.67	2.24–14.4	< 0.001	NI		
Number of Tissue biopsy	5.72	2.30–14.2	< 0.001	NI		
Detection of New metastatic organ; 0 → 4	2.35	1.39–3.97	0.001	NI		
Number of tumor lesion; < 6 vs 6 ≤	5.61	2.0–15.7	0.001	NI		
**Brain metastases; absent vs. present**	**4.40**	**1.71–11.3**	**0.002**	**27.8**	**3.13–247.8**	**0.003**
Bone metastases; absent vs. present	3.94	1.55–9.98	0.004	NI		
Number of Liquid biopsy	0.69	0.52–0.90	0.006	NI		
**Enlargement of Tumor size; < 12 mm vs 12 mm** ≤	**3.47**	**1.42–8.49**	**0.007**	**24.5**	**2.65–226.7**	**0.005**
New brain metastases; absent vs. present	4.5	1.26–16.1	0.02	NI		
Mutation site at initial diagnosis; Exon19 Deletion vs. L858R	**3.08**	**1.06–8.95**	**0.04**	3.90	0.63–24.3	0.145
**Minor site metastases; absent vs. present**	**4.20**	**1.07–16.5**	**0.04**	**21.3**	**1.40–325.6**	**0.03**
New hepatic metastases; absent vs. present	3.64	0.85–15.7	0.08	NI		
New minor site metastases; absent vs. present	4.48	0.82–24.6	0.08	NI		
Hepatic metastases; absent vs. present	2.54	0.99–1.09	0.141	NI		

Abbreviations: *OR* Odds ratio; *CI* confidence interval; Variables with a *p*-value < 0.15 on univariate analysis were entered into multivariate logistical analysis by a simultaneous method. *NI* not included in the best multivariate logistic regression model

### Positive factors associated with the T790M substitution upon liquid biopsy

Because logistic regression analysis can ascertain the degree of association between each clinical factor and the other secondary outcomes, 58 liquid biopsies were performed. The background characteristics of the group are shown in Table 7. The univariate logistic regression analyses obtained 11 variables. We also constructed four sets of multivariate models composed of variables that were not correlated with each other, as follows. Model 1 consisted of three variables, namely, mutation site at initial diagnosis, bone metastases, or detection of new tumor lesion, with an AUC of 0.936. Model 2 consisted of mutation site at initial diagnosis, enlargement of tumor size, or detection of new tumor lesion, and the AUC was 0.879. Model 3 consisted of mutation site at initial diagnosis, brain metastases, or detection of new tumor lesion, and the AUC was 0.885. Model 4 comprised mutation site at initial diagnosis, original mutation, or detection of new metastatic organ, and its AUC was 0.904. Among these models, the best model, Model 1, is shown in Table 8. Based on multivariate analysis, detecting the T790M substitution via liquid biopsy among patients harboring this mutation indicated the following: involvement of bone metastases or new tumor lesions ≥4.

**Table 7:** Background characteristics on patients harboring the T790M substitution through liquid biopsy (*n* = 58)

Variable	T790M mutation		
	Positive (*n* = 11)	Negative (*n* = 47)	Total
Original mutation			
Absent	1	38	39
Present	10	9	19
Bone metastases			
Absent	1	39	40
Present	10	8	18
Enlargement of Tumor size			
< 12 mm	7	7	14
12 mm ≤	4	40	44
Brain metastases			
Absent	4	38	42
Present	7	9	16
Detection of New tumor lesion			
< 4	4	38	42
4 ≤	7	9	16
Detection of New metastatic organ			
0	6	36	42
1	2	7	9
2	0	3	3
3	1	1	2
4	2	0	2
New minor site metastases			
Absent	8	45	53
Present	3	2	5
Hepatic metastases			
Absent	8	44	52
Present	3	3	6
Minor site metastases			
Absent	8	44	52
Present	3	3	6
Mutation site at initial diagnosis			
Exon19 Deletion	4	41	45
L858R	7	6	13
New hepatic metastases			
Absent	9	45	54
Present	2	2	4

Abbreviations: *n* number, minor site metastases; metastases of skin, kidney, ascites, lymphangiosis carcinomatosa, adrenal organ or others,

**Table 8:** Logistic regression analysis to identify patients harboring the T790M substitution through liquid biopsy (n = 58)

Variables	Univariate Analyses			Multivariate Analysis		
	OR	95%CI	*P-value*	OR	95%CI	*P-value*
Original mutation; absent vs. present	42.2	4.77–373.6	0.001	NI		
**Bone metastases; absent vs. present**	**48.8**	**5.45–436.4**	**0.001**	**77.9**	**5.32–1140**	**0.001**
Enlargement of Tumor size; < 12 mm vs 12 mm ≤	0.10	0.02–0.43	0.002	NI		
Brain metastases; absent vs. present	7.39	1.77–30.8	0.006	NI		
**Detection of New tumor lesion; < 4 vs 4** ≤	**7.39**	**1.77–30.8**	**0.006**	**14.5**	**1.38–151.2**	**0.026**
Detection of New metastatic organ; 0 → 4	1.98	1.09–3.59	0.024	NI		
New minor site metastases; absent vs. present	8.44	1.21–58.8	0.031	NI		
Hepatic metastases; absent vs. present	5.50	0.94–32.2	0.059	NI		
Minor site metastases; absent vs. present	5.50	0.94–32.2	0.059	NI		
Mutation site at initial diagnosis; Exon19 Deletion vs. L858R	3.91	0.87–17.5	0.075	1.15	0.13–10.1	0.897
New hepatic metastases; absent vs. present	5.00	0.62–40.3	0.131	NI		

Abbreviations: *OR* Odds ratio; *CI* confidence interval; Variables with a *p*-value < 0.15 on univariate analysis were entered into multivariate logistical analysis by a simultaneous method. *NI* not included in the best multivariate logistic regression model

## Discussion

Our primary purpose was to elucidate clinical features at the time of detection of T790M through clinically available mutational analysis. If we could identify these clinical features, we could perform tissue or liquid rebiopsy with more appropriate timing and reduce the frequency of tissue or liquid biopsy while maintaining the maximum DR of T790M. Accordingly, this study investigated the characteristics of *EGFR*-mutated lung cancer patients harboring the T790M substitution that was resistant to EGFR-TKIs in order to identify patients with positive features who require tissue and liquid rebiopsy. In this study, repeated biopsy revealed that 63% of *EGFR*-mutated NSCLC patients harbored the T790M substitution after acquiring clinical resistance to EGFR-TKIs. Tissue biopsy was superior to liquid biopsy in detecting T790M (*p* <  0.0001). Because liquid biopsy is a noninvasive biopsy modality for molecular-targeted analysis, including *EGFR* mutational status, it is easily reproducible using a plasma sample. The present results indicate that liquid biopsy is associated with more false-negative results in clinical practice at levels of detection of approximately 0.1–2% [14, 15]. When liquid biopsy through this test yields negative findings for the T790M substitution, it is essential to perform a tissue biopsy. Because of its high sensitivity and high DR, tissue biopsy should receive first priority.

When deciding to perform or repeat biopsy, the three relevant factors in Table 4 should be considered. We estimated the probability of detecting the T790M mutation considering the number of rebiopsies among patients with such clinical characteristics.

The timing and site are essential factors to consider for each rebiopsy (Table 6). Based on the timing of the biopsy, patients had brain metastases and minor metastases, and their tumor lesions were enlarged by > 12 mm. The enlarged tumor site would be better for tissue biopsy. When patients had bone metastases and harbored more than four new tumor lesions compared with previous tumor lesions, liquid biopsy was considered to detect the T790M substitution (Table 8).

This study has several limitations. Despite including consecutive patients herein, our study had a single-center, real-world, retrospective design. Although 405 consecutive patients were screened for the *EGFR* mutation for 3 years, they were detected in only 28% of patients (Fig. 1). Furthermore, our patient cohort comprised only 53 patients and was thus a small cohort for obtaining clinical data. Owing to remarkable progress in NSCLC treatment, we consider only minor benefits would be obtained even if we spend longer time to obtain the clinical data. In clinical practice, information from real-world data would be useful for repeated molecular analyses.

Nonetheless, this study also has some strengths. Upon testing for *EGFR*-mutant tumors by single-plexus PCR, tissue biopsy still received first priority. Because individuals with drivers receiving a matched targeted agent lived longer [16], we consider that patients with clinical characteristics similar to those harboring the T790M mutation should undergo repeated tissue or liquid rebiopsies until this mutation is detected. Furthermore, these results will help select the type or timing of biopsy.

## Conclusion

For patients with positive factors associated with the T790M substitution, this study proposes that repeated biopsy helps to maximize the DR of the T790M mutation and that rebiopsy should be repeated numerous times until this mutation is detected.

## Data Availability

All data generated or analyzed during this study are included in this published article. The datasets used and/or analyzed during the current study are available by contacting the corresponding author on a reasonable request.

## Abbreviations

NSCLC: Non-small cell lung cancer
EGFR: Epidermal growth factor receptor
TKI: Tyrosine kinase inhibitor
T790M: EGFR p.Thr790Met point mutation
VEGF: Vascular endothelial growth factor
CDx: Companion diagnostic examination
PCR: Polymerase chain reaction
PNA-LNA PCR clamp method: Peptide nucleic acid - locked nucleic acid PCR clamp method
PS: Performance status
CT: Computer tomography
ROC: Receiver-operator characteristics
DR: Detection rate
CS: Cumulative sensitivity
